# Degradation of 2,4-D by plant growth-promoting *Cupriavidus* sp. DSPFs: role in mitigating herbicide toxicity in soil and enhancing crop production

**DOI:** 10.1128/spectrum.00560-25

**Published:** 2025-09-24

**Authors:** Sandesh E. Papade, Minhaaz Suhail, Om K. Bagwe, Prashant S. Phale

**Affiliations:** 1 Department of Biosciences and Bioengineering, Indian Institute of Technology-Bombay 29491, Mumbai, India; University of Delhi, Delhi, India

**Keywords:** 2,4-dichlorophenoxyacetic acid, bioremediation, *Cupriavidus *spp., 2,4-dichlorophenol-6-monooxygenase, aromatic metabolism, phytoprotection, plant growth promotion

## Abstract

Repeated application of herbicides like 2,4-dichlorophenoxyacetic acid (2,4-D) in agriculture poses a significant environmental threat and health risk to non-target biota. *Cupriavidus* sp. strain DSPFs, isolated from Indian agricultural soil, utilizes 2,4-D and various other aromatics as the sole source of carbon and energy. It efficiently degrades high concentrations of 2,4-D (up to 0.3% wt/vol, i.e., 3,000 ppm) within 24 h with a maximum degradation rate of 105 mg L^−1^ h^−1^. Based on enzyme activity, whole-cell oxygen uptake, high-performance liquid chromatography (HPLC), and liquid chromatography-mass spectroscopy (LC-MS/MS) analyses, the strain was proposed to metabolize 2,4-D via 2,4-dichlorophenol (2,4-DCP) and 3,5-dichlorocatechol *ortho* ring-cleavage pathway. The key enzyme, 2,4-DCP monooxygenase (24DCPM), was purified to homogeneity and found to be homotetrameric with a native molecular mass of ~255 kDa with ~3.9 mol of FAD per mol of native protein. The enzyme exhibited high catalytic efficiency (*K*_cat_/*K*_m_) of 4.5 and 6.6 µM^−1^ s^−1^ with NADH and NADPH, respectively. Strain also displayed various plant growth-promoting traits like production of indoleacetic acid, ammonia, siderophores and mineral solubilization. Priming of *Vigna radiata* (mung bean) seeds with strain enhanced (30–47%) growth of seedlings in non-contaminated soil microcosms, indicating plant growth-promoting potential of the strain. Seed priming with strain significantly reduced 2,4-D toxicity to mung bean seedlings, suggesting the phytoprotective abilities of the strain. Bio-augmentation of 2,4-D-contaminated soil with strain DSPFs could remove >95% of 2,4-D (100 ppm) in 4 days with a degradation rate of 91 mg kg^−1^ day^−1^. *Cupriavidus* sp. DSPFs is a promising candidate for the eco-friendly clean-up of agricultural fields and enhancing crop productivity.

**IMPORTANCE:**

An agricultural soil isolate, *Cupriavidus* sp. strain DSPFs, is capable of degrading 2,4-dichlorophenoxyacetic acid (2,4-D), a widely used herbicide having deleterious effects on non-target crop plants and other biota. Strain DSPFs efficiently degrade relatively high concentrations of 2,4-D in minimal growth medium as well as in contaminated soil. This efficient degradation by strain can be attributed to the enhanced catalytic efficiency (low *K*_m_ and high *V*_max_) of key enzyme 2,4-dichlorophenol-6-monooxygenase (2,4-DCPM). Strain DSPFs mitigate the toxicity caused by 2,4-D to crops and also promote plant growth. This tri-functional (bioremediator-phytoprotecting-plant growth promoting) bacterium has significant potential in the eco-friendly remediation of 2,4-D in agricultural fields to prevent groundwater contamination, reduce phytotoxicity of herbicides, and enhance crop productivity.

## INTRODUCTION

Use of synthetic agrochemicals has significantly increased globally to meet the rising food demand of a rapidly expanding human population. The crop weeds, including grasses, shrubs, and climbers, often result in decreased crop yields, and their manual removal is laborious and expensive. Thus, repeated application of herbicides to control crop weeds has become an inevitable process in both agricultural production and gardening (1). 2,4-Dichlorophenoxyacetic acid (2,4-D) is the most widely applied organic, phenoxyacid derivative herbicide that is selectively used against broad-leaved weeds in agricultural as well as non-agricultural soils. The global annual application of 2,4-D is estimated to be 45,000 to 90,000 tons, which is only predicted to increase further (2, 3). Persistence of applied 2,4-D (up to 312 days) in soil increases the chances of leaching and has been detected in ground as well as surface water worldwide, posing environmental risk (4–8). Even at low concentrations, 2,4-D and its metabolites, such as 2,4-dichlorophenol, show genotoxic, cytotoxic and mutagenic effects in plants as well as significant histological, physiological and behavioral alterations in fishes, lower mammals and humans (3, 9–11). Hence, complete removal of these pollutants through cost-effective and eco-friendly strategies like bioremediation is of prime importance.

**Fig 1:**
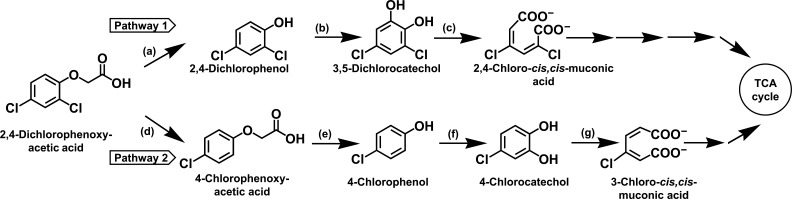
Metabolic pathways for degradation of 2,4-dichlorophenoxyacetic acid (2,4-D) in bacteria. Enzymes involved are (**a**) α-ketoglutarate-dependent 2,4-D dioxygenase; (**b**) 2,4-dichlorophenol-6-monooxygenase; (**c**) 3,5-dichlorocatechol 1,2-dioxygenase; (**d**) 2,4-D dehalogenase; (**e**) 4-chlorophenoxyacetate monoxygenase; (**f**) 2,4-dichlorophenol-6-monooxygenase; and (**g**) 4-chlorocatechol 1,2-dioxygenase.

2,4-D degrading bacterial strains belonging to genera *Pseudomonas*, *Arthrobacter*, *Corynebacterium*, *Achromobacter*, *Cupriavidus*, *Flavobacterium*, and *Streptomyces* have been isolated from diverse ecological niches (11–15). The metabolism of 2,4-D in bacteria typically proceeds via the 2,4-dichlorophenol (2,4-DCP) route (pathway 1; Fig. 1). 2,4-Dichlorophenol-6-monooxygenase (24DCPM) catalyzes the conversion of 2,4-DCP to 3,5-dichlorocatechol (35DCC), which is further *ortho*-ring cleaved by chlorocatechol-1,2-dioxygenase (CC12DO) to metabolic intermediates that are funneled into the central carbon cycle (16, 17). Alternatively, bacteria like *Azotobacter chroococcum* dechlorinate 2,4-D to 4-chlorophenoxyacetic acid (4-CPA), which is then converted to 4-chlorophenol (4-CP) by the action of 4-CPA monooxygenase (pathway 2; Fig. 1). Furthermore, 4-CP is subsequently catabolized by 24DCPM and CC12DO to central carbon pathway intermediates (13, 18).

The slow rate of bacterial degradation under both laboratory and environmental conditions is the major limitation in the bioremediation of 2,4-D (12, 14, 19, 20). For example, *Cupriavidus* sp. CY-1 is reported to degrade 2,4-D (0.05% wt/vol, i.e., 500 ppm); however, complete degradation was achieved by 72 h with maximum growth of 0.2, OD_600nm_ (21). *Cupriavidus campinensis* takes around 96 h for complete degradation of 0.07% 2,4-D (22). Comparatively, at higher concentrations, i.e., 0.1% and 0.3% of 2,4-D, *Cupriavidus oxalaticus* X32 degrades only 75% and 20% of 2,4-D, respectively, by 72 h (20). Catalytic bottleneck in the 2,4-D metabolism plays a significant role in limiting its degradation. 2,4-DCP is formed as a metabolic intermediate during 2,4-D degradation and is more toxic than 2,4-D itself. Notably, 24DCPM is a key mono-oxygenase involved in both degradation pathways, catalyzing the conversion of toxic intermediates into less toxic products (2,4-DCP → 3,5-DCC in pathway 1 and 4-CP → 4-chlorocatechol in pathway 2; Fig. 1). It is crucial to maintain a balance in the rate of reaction, i.e*.*, turnover rate of 24DCPM for the survival of 2,4-D degrading bacteria. Any imbalance or low turnover rate can impair bacterial growth and degradation capacity or even result in cell death (23–25).

Bioaugmentation of agricultural sites with microorganisms is considered to be an efficient, cost-effective and environmentally friendly approach for mitigating herbicide pollution and its phytotoxicity (26, 27). Various studies have shown that 2,4-D-degrading strains hold potential for bioremediation of 2,4-D contaminated sites (19–21). However, limited research has focused on using microorganisms to remove 2,4-D from soil, mitigate the phytotoxicity to crops caused by 2,4-D residues, and its plant growth-promoting activity (16).

The present study aimed to isolate and characterize efficient 2,4-D-degrading bacteria from agricultural soil, with a focus on its potential for bioremediation and plant growth promotion. Objectives are to evaluate the degradation efficiency of the isolate under *in vitro* and soil conditions, catalytic efficacy of key enzymes involved in 2,4-D metabolism, investigate the plant growth-promoting traits, and the ability to mitigate 2,4-D toxicity to crop plants. By integrating microbiological, biochemical, and plant-based assays, the study explores the multifaceted potential of the selected strain for sustainable remediation of herbicide-contaminated soils and improved agricultural productivity.

## MATERIALS AND METHODS

### Bacterial strain and culture conditions

#### Isolation and identification of 2,4-D degrading bacteria

Soil samples (~1 kg) were collected from agricultural farms with a history of repeated application of 2,4-D, located at Junnar tehsil of Pune district, India (19°13′00.3″N 73°51″′43.8″E). 2,4-D degrading bacteria were isolated from soil (~2 g) using enrichment culture technique in 150 mL minimal salt medium (MSM, pH 7.5; [L^−1^]: K_2_HPO_4_, 8 g; KH_2_PO_4_, 1 g; NH_4_Cl, 1 g; MgSO_4_.7H_2_O, 100 mg; MnSO_4_.H_2_O, 1 mg; CuSO_4_.5H_2_O, 1 mg; FeSO_4_.7H_2_O, 5 mg; H_3_BO_3_, 1 mg; CaCl_2_.2H_2_O, 1 mg, and NaMoO_4_, 1 mg) (28) supplemented with 2,4-D (0.1%, wt/vol) in 500 mL capacity baffled Erlenmeyer flasks at 30°C on a rotary shaker (200 rpm). Enriched culture was plated on MSM plus 2,4-D agar plate, and the pure bacterial culture was raised from a single isolated colony. The culture was designated as strain DSPFs.

The 16S rRNA gene was amplified from the genome of strain DSPFs using universal primers 27F and 1492R (29), and the amplicon (~1.5 kb) was sequenced (Eurofins Genomics India Pvt. Ltd., India). A comparative phylogenetic tree was constructed with its closely related validly named type species [retrieved from EzBiocloud (http://www.ezbiocloud.net/)] using the model of neighbor-joining method in MEGA 11 software with 1,000 bootstrap replicates.

#### Growth profile, degradation kinetics and stability of phenotype

Strain DSPFs was cultured/grown on MSM supplemented with 2,4-D (0.1%, wt/vol), and growth was monitored by measuring the optical density at 540 nm, using a spectrophotometer (PerkinElmer, Lambda 35). 2,4-D degradation rates for strain DSPFs were determined by estimating the residual amount of 2,4-D in the spent medium at regular time intervals using high-performance liquid chromatography (HPLC). The percentage and rate of 2,4-D degradation were calculated as follows:

Degradation (%) = [(C_0_ − C_t_)/C_0_] × 100,

Rate of degradation (mg L^−1^ h^−1^) = (C_0_ − C_t_)/t.

Where t is the culture time while C_0_ and C_t_ are the concentrations (mg L^−1^) of 2,4-D at time 0 and t (h), respectively.

The stability of the 2,4-D degradation property was determined as described earlier (30). Briefly, strain DSPFs was grown on Luria-Bertani (LB) medium with or without curing agents (10, 25, 50 µg mL^−1^ of ethidium bromide or acridine orange) for five transfers (each transfer after 14 h). Appropriate dilutions were plated onto Luria agar plates. Isolated colonies were replica plated on MSM plus 2,4-D (0.1%) agar plates, scored for growth (48–72 h at 30°C), and reported as percent stable for 2,4-D degradation property.

### Metabolic studies

#### Whole-cell oxygen uptake

Cells of strain DSPFs grown on 2,4-D (0.1%, wt/vol) or fructose (0.25%, wt/vol) were used to monitor the whole-cell O_2_ uptake. The respiration rates were measured in the presence of various probable metabolic intermediates at 30°C using Oxygraph (Hansatech, UK) fitted with Clark’s O_2_ electrode as described previously (31).

#### Enzyme assays

The activity of 24DCPM was monitored spectrophotometrically (PerkinElmer, Lambda 35) by measuring the rate of decrease in the absorbance at 340 nm due to disappearance of NAD(P)H (ε_340_ = 6,220 M^−1^ cm^−1^ [23]); or polarographically by measuring the rate of consumption of oxygen (Oxygraph, Hansatech, UK (32). The reaction mixture (1 mL) contained potassium phosphate buffer (50 mM, pH 7.5, hereafter referred to as phosphate buffer), FAD (6.25 µM), 2,4-dichlorophenol (2,4-DCP; 50 µM), NADH (200 µM), or NADPH (300 µM) and an appropriate amount of the cell-free extract (CFE)/enzyme. One unit of 24DCPM activity was defined as micromoles of NAD(P)H or oxygen consumed per minute per milliliter of enzyme. Specific activity was defined as micromoles of NAD(P)H or oxygen consumed per minute per milligram of protein.

3,5-Dichlorocatechol 1,2-dioxygenase (35DCC12DO) activity was monitored by measuring the rate of increase in absorbance at 268 nm due to formation of reaction product (2,4-dichloro-*cis,cis*-muconic acid) at 268 nm (ε_268_ = 13,400 M^−1^ cm^−1^ [33]). The reaction mixture (1 mL) contained 3,5-dichlorocatechol (100 µM), an appropriate amount of CFE, and phosphate buffer. One unit of 35DCC12DO activity was defined as nanomoles of product formed per minute per milliliter of enzyme. Specific activity was defined as nanomoles of product formed per minute per milligram of protein.

The protein estimation was performed as described by Bradford (34) using bovine serum albumin (BSA) as a standard.

#### Identification of metabolic intermediates

The metabolites of 2,4-D degradation by strain DSPFs were extracted from the acidified spent medium as well as from cell-free extract (CFE, see below) in equal volumes of ethyl acetate and concentrated. The metabolites were also resolved and identified by high-performance liquid chromatography (HPLC; Jasco, LC-4000; equipped with photodiode array detector) using octadecylsilane silica (C_18_) reverse-phase column (150 × 4.6 mm) and methanol: *ortho*-phosphoric acid (0.1%), 60:40 (vol/vol) as mobile phase at a flow rate of 1 mL min^−1^ (12). Metabolites were detected at 230 nm, identified by comparing the retention time (RT), and quantified using respective concentration versus peak area plots for the standards. Metabolites were further confirmed by liquid chromatography-mass spectroscopy (LC-MS/MS; 1290 Infinity UHPLC, Agilent, USA) at SAIF, IIT-Bombay. The instrument was configured with an autosampler (HiP, Agilent), a binary HPLC pump (Agilent 1100, with microvacuum degasser), and a triple quadrupole mass spectrometer (G6550A, with Dual AJS ESI). The gradient elution with formic acid (1% vol/vol in water) and methanol was used with a constant flow rate of 0.3 mL min^−1^.

### Purification of 2,4-dichlorophenol-6-monooxygenase

#### Preparation of cell-free extract

Cells grown on 2,4-D (0.2%, wt/vol) till late log phase were harvested, washed, re-suspended in phosphate buffer, and disrupted by sonication on ice (Five cycles with 5 min interval; each cycle: 15 pulses, output 11 W, Ultrasonic processor GE130). The cell lysate was centrifuged at 30,000 × *g* for 30 min (4°C). The clear supernatant obtained was referred to as CFE, which was centrifuged at 100,000 × *g* for 1 h at 4°C to obtain membrane-free CFE and used for further enzyme purification. All purification steps were carried out at 4°C.

#### Ammonium sulfate fractionation

The membrane-free CFE was brought to 0–30%, followed by 30–60% saturation by gradual addition of solid ammonium sulfate. The suspension was incubated on ice with constant stirring and centrifuged at 20,000 × *g* for 20 min at 4°C. The pellet obtained was dissolved in a minimum volume (~1 mL) of phosphate buffer and dialyzed for 16 h against two changes of 1 L of the same buffer at 4°C. The dialyzed 30–60% ammonium sulfate fraction showed 24DCPM enzyme activity.

#### Purification by affinity and size exclusion chromatography

The 2,4-DCP-coupled Sepharose CL-4B affinity matrix was prepared as described by Radjendirane et al. (32) (Fig. S1). The dialyzed 30–60% ammonium sulfate fraction was loaded onto an affinity matrix (total column volume, 10 mL; height, 9 cm; bed volume, 5 mL) pre-equilibrated with phosphate buffer. The matrix was washed thoroughly with the same buffer (2 CV). The bound 24DCPM was eluted using 5 mM 2,4-DCP in phosphate buffer. 24DCPM moved as a yellow band, and fractions (0.5 mL) were collected at a flow rate of 20 mL h^−1^. Active and pure fractions (as determined by enzyme activity and SDS-PAGE) were pooled, concentrated using Centricon (30 K, Pall corporation), and applied to size-exclusion chromatography (ENrich SEC 650 column, Bio-Rad, CV = 24 mL, pre-equilibrated and developed with phosphate buffer). The fractions (0.5 mL) were collected at a flow rate of 30 mL h^−1^ using phosphate buffer. The active and pure fractions were pooled and used for further analysis.

The purity of enzyme preparations was assessed using SDS-PAGE (35) and native-PAGE (36). Subunit molecular mass was determined by SDS-PAGE using molecular weight markers (kDa): lysozyme (14.3), soybean trypsin inhibitor (20.1), carbonic anhydrase (29), ovalbumin (43), bovine serum albumin (66), and phosphorylase b (97.4). The native molecular mass of the enzyme was determined by size-exclusion chromatography. The column was calibrated with standard protein molecular mass markers (kDa): cytochrome c (12.4), carbonic anhydrase (29), BSA (66), alcohol dehydrogenase (150), β-amylase (200), apoferritin (443), and thyroglobulin (669). The molecular mass of 24DCPM was determined from a plot of log (mol mass) versus *V*_elution_ /*V*_void_ (*V*_e_/*V*_0_).

#### Spectroscopic characterization of 24DCPM

The UV-visible absorption spectra of purified 24DCPM (1 µM) were recorded in the range of 200–700 nm in phosphate buffer using a spectrophotometer (PerkinElmer, Lambda 35). The emission spectra (450–600 nm) of 24DCPM (1 µM) were recorded by exciting it at 445 nm using a fluorescence spectrometer (Jasco FP8500).

Far-UV (198–260 nm) circular dichroism (CD) spectra of 24DCPM (1 µM, in 0.2 cm path length cuvette, 25°C) in phosphate buffer were recorded using a spectropolarimeter (Jasco-J-1500). All spectra were corrected against respective baseline spectra. The percent secondary structure was predicted using the online Bestsel server (https://bestsel.elte.hu/index.php).

#### Determination of kinetic constants

To determine *K*_m_ and *V*_max_, the initial reaction rates were determined by varying the concentration of 2,4-DCP (2.5–250 µM) in the presence of a fixed concentration of NADH (200 µM) or NADPH (300 µM) by spectrophotometric as well as polarographic methods as described earlier. Similarly, *K*_m_ and *V*_max_ for NADH and NADPH were determined by varying the concentration of the respective cofactor (10–1200 µM) in the presence of a fixed concentration of 2,4-DCP (50 µM). For 2,4-DCP, the data were fitted using uncompetitive substrate inhibition to *v* = *V*_max_ [*S*]/(*K*_m_ + [*S*]) (1 + ([*S*]/*K*_i_), and for NADH and NADPH, the Michaelis-Menten equation *v* = *V*_max_ [*S*]/(*K*_m_ + [*S*]) was followed. In the equation, *v* and *V*_max_ are the initial and maximum velocity, [*S*] is the substrate concentration, *K*_m_ and *K*_i_ are the Michaelis-Menten and inhibition constant, respectively.

### Microcosm studies

#### Release of 2,4-D phytotoxicity to *Vigna radiata* by strain DSPFs

The microcosm assay was performed to assess the ability of strain DSPFs to mitigate toxicity caused by 2,4-D to mung bean (*Vigna radiata*). Microcosms were prepared in pots (150 mL capacity) containing dry, non-sterile, agricultural soil (100 g; clay, 60.9%; silt, 29.9; sand, 9.1%; organic content, 7.9%; carbon, 2.7%; nitrogen, 0.04; specific gravity, 2.4). Soil was spiked either with 25, 50, 100, 250, or 500 ppm of 2,4-D or left unamended. Mung bean seeds were surface sterilized and bacterized with benzoate (0.1%, wt/vol), grown cells of strain DSPFs (10^8^ CFU mL^−1^) as described (37). Seeds primed with/without strain DSPFs were sown in pots spiked with/without 2,4-D. Pots were kept at controlled laboratory conditions (25°C, 12 h day-night cycle) and seed germination was monitored every 24 h for 10 days. On the 10th day, seedlings were analyzed for shoot and root length, wet and dry biomass. An experiment was performed in duplicates (two microcosms with 10 seeds each) and repeated at least three times, independently.

#### Bioremediation of 2,4-D-contaminated soil by strain DSPFs

Bioremediation experiments were performed in microcosms containing dry, non-sterile agricultural soil (100 g; as mentioned above) spiked with 2,4-D (100 mg kg^−1^ of soil, i.e., 100 ppm). Soil in the microcosms was inoculated (bioaugmented) with/without strain DSPFs (10^7^ CFU g^−1^ of soil) and sown with/without mung bean seeds. The moisture content of the soil was adjusted with water to 20–30%. Pots were kept at controlled laboratory conditions (25°C, 12 h day-night cycle) for 10 days, and soil samples were collected every 24 h. 2,4-D was extracted from a 1 g dry soil sample in 1.5 mL solvent (1:1 vol/vol, water:acetonitrile) and quantified using HPLC. A recovery percentage of 2,4-D from soil was 96% ± 2%. The rate of 2,4-D degradation by strain DSPFs in microcosms was calculated as follows:

Rate of degradation (mg kg^−1^ day^−1^) = (C_0_ − C_t_)/t.

Where t is the incubation time and C_0_ and C_t_ are the concentration (mg kg^−1^) of 2,4-D at time 0 and t (day), respectively.

#### Assessment of plant growth-promoting traits

Strain DSPFs was assessed for various plant growth-promoting (PGP) traits like inorganic minerals (phosphate, potassium) solubilization, production of indoleacetic acid (IAA), siderophores, ammonia, and 1-aminocyclopropane-1-carboxylate (ACC) deaminase as described previously (37). Briefly, phosphate solubilization ability was qualitatively analyzed in Pikovskaya’s broth medium supplemented with tri-calcium phosphate (38). Potassium solubilization was examined on Aleksandrov agar medium with potassium aluminosilicates (Feldspar [39]). IAA production in the presence or absence of L-tryptophan was estimated by Salkowski’s method (40). The ability of strain DSPFs to produce siderophores was quantified in King’s B medium using CAS reagent (41). Ammonia production in peptone water broth was quantified using Nessler’s reagent (42). ACC deaminase production ability was assessed in Dworkin and Foster salt minimal medium supplemented with ACC (3 mM) as the sole source of nitrogen (43).

### Statistical analysis

All experiments were performed at least three times independently in triplicate. Arithmetic averages and standard deviations were calculated and analyzed further. The data were calculated and analyzed with the SigmaPlot software (version 12.3) and/or GraphPad Prism software (version 10). The mean difference comparison between the treatments was analyzed by analysis of variance and subsequently by Tukey’s post hoc analysis. The statistical significance was determined at *P* < 0.05. The letter “n” denotes sample size (biological replicates) and the letter “N” denotes number of population size, i.e*.*, the total number of observations (44).

## RESULTS AND DISCUSSION

### Isolation, identification and growth characterization of 2,4-D degrading strain DSPFs

**Fig 2:**
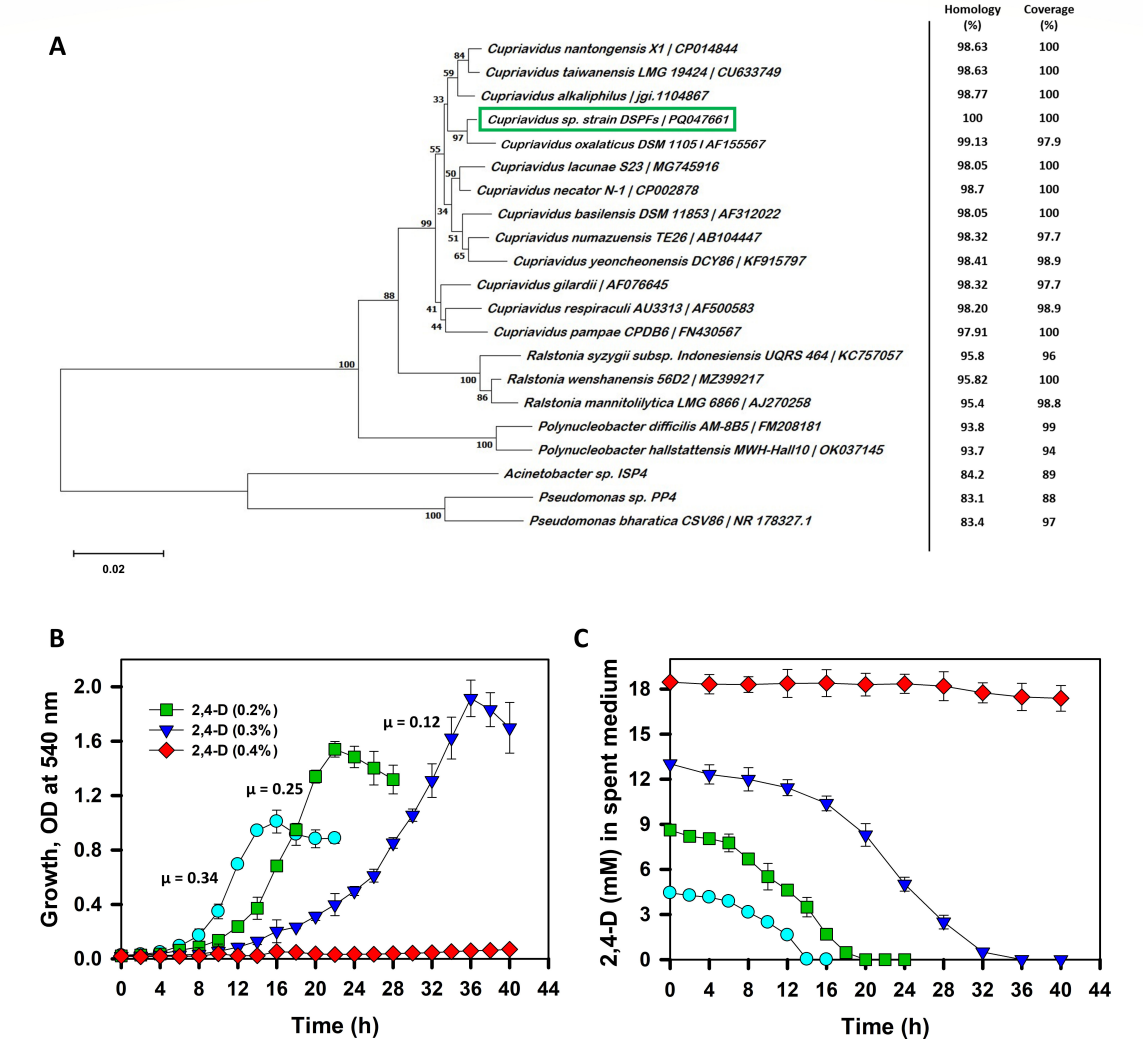
Taxonomic identification and growth properties of *Cupriavidus* sp. strain DSPFs. (**A**) represents comparative phylogenetic analysis of strain DSPFs based on the 16S rRNA gene sequence. The scale indicates the percent of nucleotide substitution, while numbers near corresponding branches indicate the bootstrap value (>50%). (**B**) depicts the growth profile of strain DSPFs on 0.1% (wt/vol; 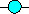), 0.2% (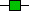), 0.3% (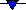), and 0.4% (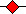) of 2,4-D as the sole carbon source. The specific growth rate, μ (h^−1^), is depicted. (**C**) represents residual 2,4-D concentration in the spent medium from 0.1% (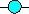), 0.2% (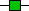), 0.3% (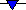), and 0.4% (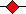) of 2,4-D-grown culture. Experiments were performed independently at least three times, and data are presented as the arithmetic mean with standard deviation.

Three 2,4-D-degrading bacterial strains, designated as DSPFs, DPTF1 and DPTF2, were isolated from two agricultural soil samples using an enrichment culture technique with 2,4-D as the sole source of carbon and energy. Among the three isolates, strain DSPFs was selected for further study due to its ability to degrade 2,4-D within 16 h. DSPFs is a Gram-negative, aerobic, motile, and rod-shaped bacterium. It showed a positive reaction for oxidase, catalase test, and metabolized malate, citrate, fructose, glycerol, while failed to metabolize glucose, galactose, arabinose, mannitol, maltose, and sucrose as carbon sources (Table S1; Supplementary material). Based on 16S rRNA gene sequencing and its phylogenetic analysis, strain DSPFs was identified as *Cupriavidus* sp. (GenBank accession number PQ047661, Fig. 2A).

The tolerance and degradation capacity of bacteria decrease at higher concentrations of pesticides like 2,4-D (26, 45). To study the effect of various concentrations of 2,4-D on biodegradation and growth pattern, strain DSPFs was grown in MSM (pH 7.5 at 30°C) containing 2,4-D at 0.1–0.4 %, wt/vol (i.e., 1,000–4,000 ppm or mg L^−1^). A long lag phase with low specific growth rate was observed at higher concentrations of 2,4-D, and the strain failed to grow at 0.4% of 2,4-D (Fig. 2B). The residual concentration of 2,4-D in growth medium reached zero by 16, 22, and 36 h (Fig. 2B and C) with degradation rates of 71, 105, and 90 mg L^−1^ h^−1^ for 0.1%, 0.2%, and 0.3% of 2,4-D, respectively, at the same cell density (OD_540_ = ~1; 3 × 10^9^ cells mL^−1^). These results indicate that strain DSPFs has the ability to tolerate as well as degrade 2,4-D at higher concentrations (up to 0.3%, wt/vol) efficiently and has better potential for bioremediation application compared to previously reported strains (26, 46).

### Elucidation of the metabolic pathway for 2,4-D degradation

**Fig 3:**
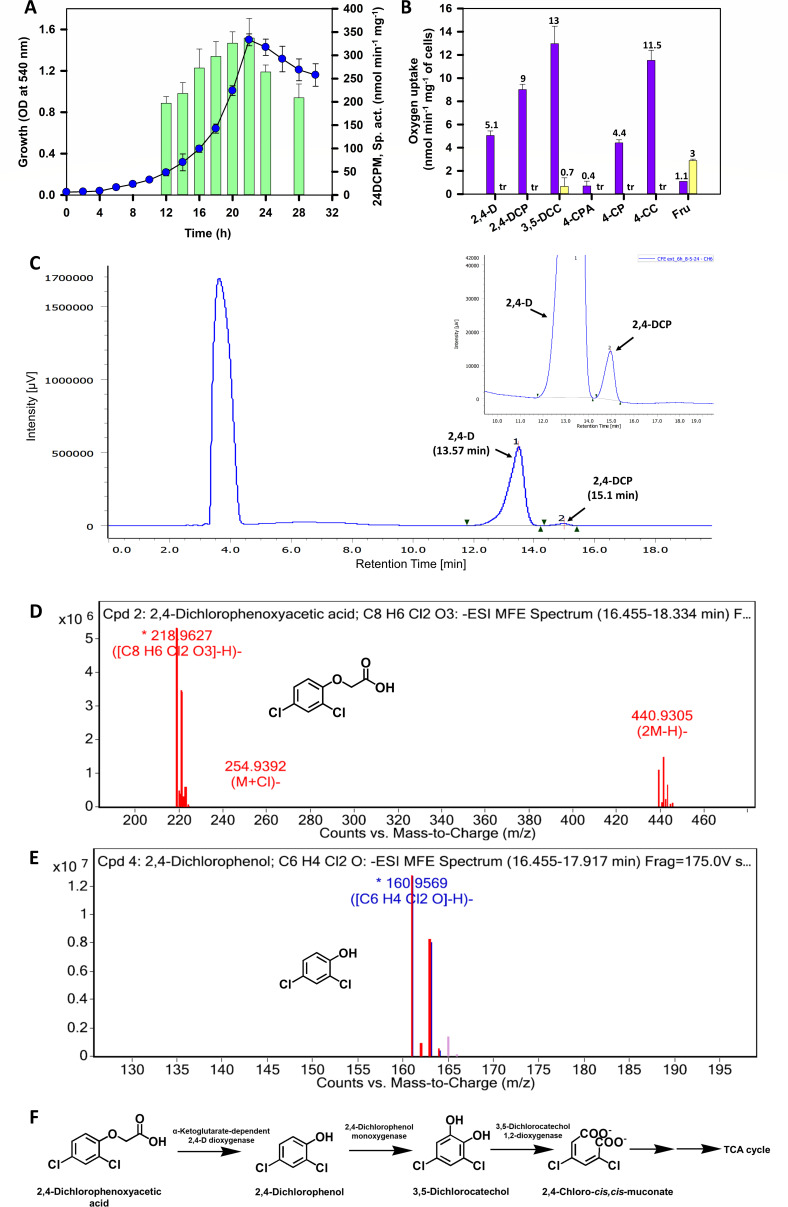
Elucidation of metabolic pathway for 2,4-dichlorophenoxyacetic acid (2,4-D) in *Cupriavidus* sp. strain DSPFs. (**A**) depicts the growth profile (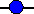) of strain DSPFs on 2,4-D (0.2%, wt/vol) and specific activity profile (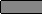) of 2,4-dichlorophenol-6-monooxygenase (24DCPM). (**B**) represents whole-cell oxygen uptake rates for cells grown on 2,4-D (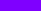) or fructose (Fru; 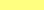) on 2,4-D, 2,4-dichlorophenol (2,4-DCP), 3,5-dichlorocatechol (3,5-DCC), 4-chlorophenoxyacetic acid (4-CPA), 4-chlorophenol (4-CP), and 4-chlorocatechol (4-CC). (**C**) depicts HPLC analysis of metabolites extracted from CFE of cells grown on 2,4-D (0.2%). The peaks and the retention times of corresponding metabolites have been indicated on the chromatograms. Inset, expanded view of chromatogram showing the distinct peak of metabolite 2,4-DCP. (**D**) and (**E**) represent LC-MS/MS analysis of CFE of cells grown on 2,4-D (0.2%, wt/vol). Mass spectra depict the presence/detection of 2,4-D (**D**) and 2,4-DCP (**E**). (**F**) represents the proposed pathway for the degradation of 2,4-D in strain DSPFs.

In several bacteria, including *Cupriavidus* spp., 2,4-D is metabolized via 2,4-DCP to 3,5-dichlorocatechol (3,5-DCC) by employing enzyme 2,4-dichlorophenol-6-monooxygenase (24DCPM; Pathway 1; Fig. 1) (13). CFE prepared from 2,4-D grown cells of DSPFs showed activity of 24DCPM (catalyzes the conversion of 2,4-DCP to 3,5-DCC) and 3,5-dichlorocatechol-1,2-dioxygenase (35DCC12DO; catalyzes the conversion of 3,5-DCC to 2,4-dichloro-*cis,cis*-muconate). Specific activities of 24DCPM (Fig. 3A) and 35DCC12DO (Fig. S2; Supplementary material) increased during the log phase of growth, reaching a maximum in the late log phase. Cells grown on 2,4-D showed significant oxygen uptake in the presence of 2,4-D, 2,4-DCP, and 3,5-DCC but negligible (<0.5 nmol min^−^¹ mg^−^¹) oxygen uptake with 4-CPA (Fig. 3B). The respiration observed with 4-CP and 4-CC could be due to the wide-substrate specificity of 24DCPM and chlorocatechol-1,2-dioxygenase of pathway 1 (18). Fructose-grown cells exhibited negligible oxygen uptake on these compounds (Fig. 3B), indicating the inducible nature of 2,4-D metabolism. 2,4-DCP and 3,5-DCC could not be detected in the spent medium of culture. Detection of 2,4-DCP by HPLC in the ethyl acetate extract of CFE prepared from 2,4-D grown cells and its further mass analysis by LC-MS/MS suggests 2,4-DCP as a probable metabolic intermediate (Fig. 3C through E).

Strain DSPFs showed complete degradation of 2,4-DCP and 3,5-DCC (0.001%, i.e., 10 ppm) by 16 and 12 h with maximum growth (OD_540_) of 0.26 and 0.29, respectively. These results indicate the ability of the strain to metabolize 2,4-DCP and 3,5-DCC. Based on enzymatic assays, whole-cell oxygen uptake, and metabolic analysis, strain DSPFs was proposed to metabolize 2,4-D via the 2,4-DCP → 3,5-DCC → 2,4-dichloromuconic acid pathway (Fig. 3F). A similar metabolic pathway is reported in *Cupriavidus gilardii* T1 (26). *Achromobacter xylosoxidans* EST4002 and *Cupriavidus necator* JMP134 harboring *tfd*ABCDEF genes encode several 2,4-dedegrading enzymes and metabolize 2,4-D to 3,5-DCC, which is *ortho* ring-cleaved to 2,4-dichloromuconate and then funneled to the tricarboxylic acid (TCA) cycle (47, 48).

In various bacteria, including *A. xylosoxidans* EST4002 and *C. necator* JMP134, capable of 2,4-D degradation, *tfd* genes are reported to be present on the plasmid DNA that can be cured/lost under various stress conditions, like in the absence of selection pressure (49). To investigate the stability of the 2,4-D degradation phenotype in strain DSPFs, cells were grown in the presence of curing agents like acridine orange/ethidium bromide, which have been reported to cure plasmid(s) involved in the aromatic-degrading microorganisms (50, 51). All screened colonies (3,216) of strain DSPFs showed the ability to degrade 2,4-D even after being treated with high concentration (50 µg mL^−1^) of curing agents, suggesting a stable degradation phenotype probably encoded by genes present on the chromosome or stable plasmid DNA (Table S2; Supplementary material). The stable and non-curable 2,4-D degradation property confers an advantage to DSPFs to maintain the degradation phenotype even in the absence of selection pressure, 2,4-D. Such strains have better survivability in nature and are more suitable for the bioremediation of the contaminated environment and agricultural fields.

### Ability to degrade a broad range of aromatics and metabolic diversity

Besides 2,4-D, strain DSPFs metabolizes phenylacetate, hydroxyphenylacetate, phthalate, isophthalate, 2-/4-nitrobenzoate, benzyl alcohol, benzaldehyde, ferulic acid, vanillin, and vanillic acid amongst other aromatics as the sole source of carbon and energy. Metabolic and genomic versatility of the *Cupriavidus* genus is well reported, with the ability to metabolize a diverse array of aromatic compounds (47, 52). Microbial degradation of aromatics typically proceeds through common intermediates such as benzoate or hydroxybenzoates, which are then ring-cleaved to central carbon metabolites via catechol or dihydroxybenzoates (protocatechuate or gentisate). The bacterial degradation of benzoate and salicylate has been documented (53, 54). However, the degradation of benzoate, 2-hydroxybenzoate (salicylate), and 3-/4-hydroxybenzoate by a single strain, especially the aerobic degradation of 3-hydroxybenzoate, has rarely been reported (47, 55). Strain DSPFs could efficiently metabolize benzoate and all three hydroxybenzoate isomers as the sole source of carbon and energy (Fig. S3; Supplementary material). The growth of strain DSPFs on benzoate was slower (specific growth rate, µ = 0.2 h^−1^). However, it showed rapid growth on 3-hydroxybenzoate (3-HBA; µ = 0.49 h^−1^) followed by salicylate (µ = 0.41 h^−1^) and 4-hydroxybenzoic acid (4-HBA; µ = 0.4 h^−1^), reaching the stationary phase within 12 h (Fig. S3; Supplementary material).

CFE prepared from DSPFs cells grown on benzoate and 4-HBA showed activity of catechol 1,2-dioxygenase and protocatechuate 3,4-dioxygenase, respectively (Table S3; Supplementary material). The CFE prepared from salicylate and 3-HBA-grown cells showed the presence of gentisate 1,2-dioxygenase activity. These results suggest that strain DSPFs possess metabolic (lower/downstream) pathways for degradation of all three central intermediates (i.e., catechol, protocatechuate, and gentisate) in aerobic degradation of aromatics, highlighting metabolic diversity. This makes strain DSPFs a potential host for engineering upper metabolic routes to achieve complete degradation of diverse aromatic pollutants. Moreover, the ability of strain DSPFs to degrade 2,4-D as well as other aromatic compounds indicates its potential in environmental restoration, particularly in agricultural settings where herbicide contamination is prevalent, with the presence of other aromatics (applied as pesticides or stabilizers in agrochemicals).

### Purification and spectroscopic characterization of key enzyme 2,4-dichlorophenol-6-monooxygenase

2,4-DCP is a key metabolic intermediate formed during 2,4-D degradation and is also commonly used in industrial and agricultural applications, such as pesticides and germicides (56). It is more toxic and recalcitrant than 2,4-D. The enzyme 24DCPM plays a crucial role in the degradation of 2,4-D by catalyzing the conversion of 2,4-DCP to the less toxic 3,5-DCC. Strain DSPFs tolerates high concentrations of 2,4-D and effectively metabolizes with a high degradation rate (~70–90 mg L^−1^ h^−1^) (Fig. 2B and C; Fig. 3F), as compared to reported bacteria (26, 46). To understand the probable role of enzyme 24DCPM in the efficient degradation of 2,4-D, 24DCPM from strain DSPFs was purified and characterized.

**Fig 4:**
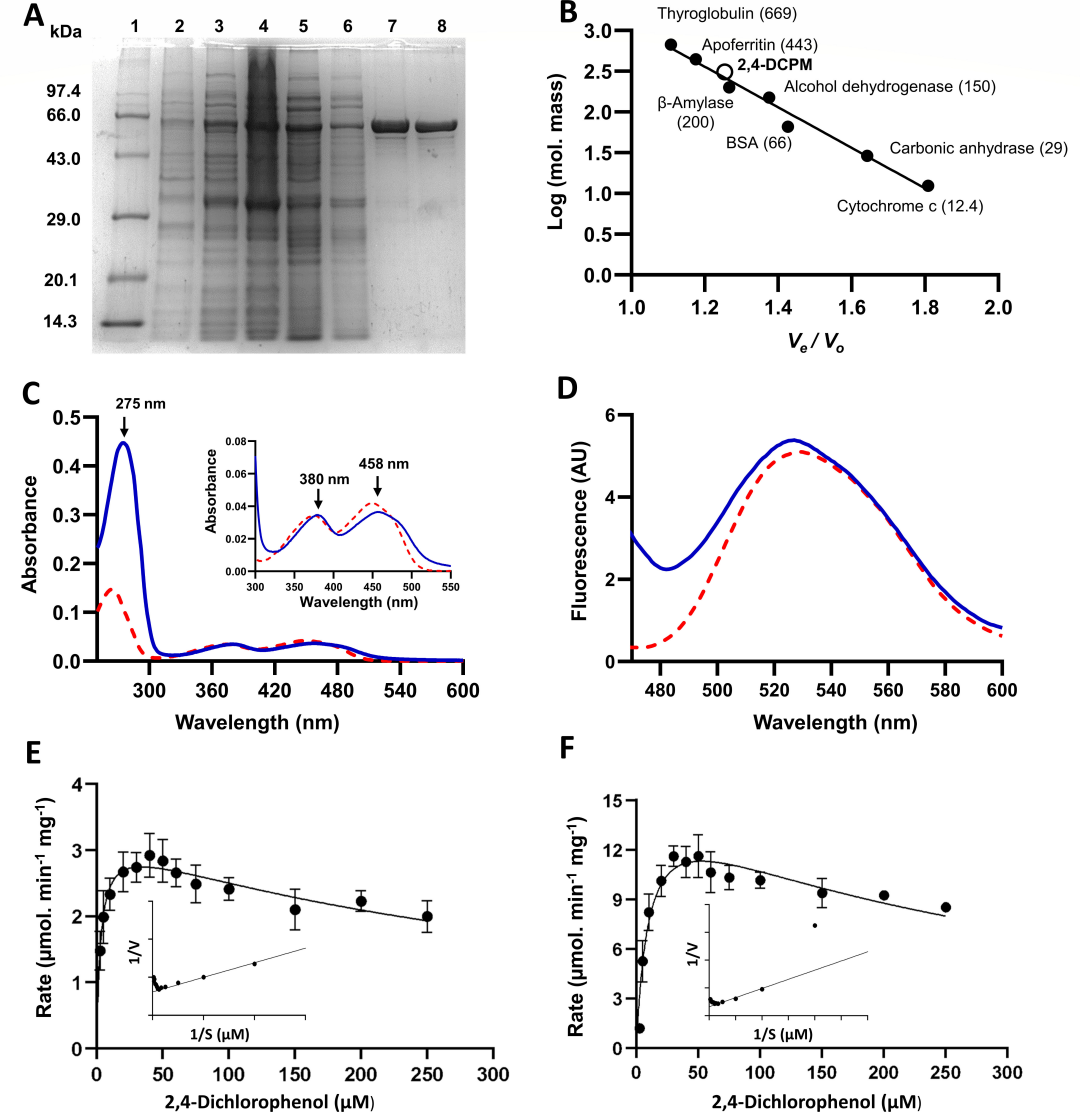
Purification and characterization of 24DCPM from *Cupriavidus* sp. strain DSPFs. (**A**) depicts SDS-PAGE analysis of 24DCPM during different stages of protein purification. *Lane 1*, molecular mass marker (kDa): phosphorylase B (97.4), BSA (66), ovalbumin (43), carbonic anhydrase (29), soybean trypsin inhibitor (20.1), and lysozyme (14.3); *lane 2*, pellet fraction; *lane 3*, cell-free extract; *lane 4*, 30–60% ammonium sulfate saturation fraction; *lane 5*, unbound fraction from affinity chromatography; *lane 6*, wash fraction from affinity chromatography; *lane 7*, affinity purification pool; *lane 8*, gel filtration chromatography (each lane contains 25 µg of protein). (**B**) represents the plot of log (molecular mass) versus *V*_e_/*V*_0_ for gel filtration standard molecular mass protein markers, represented by filled circles. The open circle represents 24DCPM. (**C**) depicts UV-visible absorption spectra of native 24DCPM (1 µM; solid line) and authentic FAD (62.5 nM; dotted line) in potassium phosphate buffer (50 mM, pH 7.5). Inset, magnified view of the visible absorption spectra of 24DCPM. (**D**) depicts the fluorescence emission spectra of native FAD-bound 24DCPM (1 µM, solid line) and authentic FAD (6.25 nM, dotted line) excited at 445 nm in phosphate buffer (50 mM, pH 7.5). (**E**) and (**F**) represent kinetic properties of 24DCPM: plots of initial velocity (V) versus [*S*] for 24DCPM with NADH (**E**) or NADPH (**F**) as cofactor.

24DCPM was purified using ammonium sulfate fractionation, 2,4-DCP-Sepharose CL-4B affinity chromatography, and ENrich SEC 650 gel filtration chromatography (Fig. 4A). The enzyme was purified to apparent homogeneity with a 15.4-fold increase in specific activity of 2.9 µmol min^−^¹ mg^−^¹ and yield of 6.2% (Table 1). Gel filtration and SDS-PAGE analysis indicate that 24DCPM is a homotetramer with a native molecular mass of ~255 kDa and subunit mass of ~65 kDa (Fig. 4A and B). Enzyme displayed maximum activity in phosphate buffer (50 mM) at pH 7.5 and was stable (retained about ~100% activity) after 10 days of storage at 4°C.

**TABLE 1:** Purification summary of 2,4-dichlorophenol-6-monooxygenase from *Cupriavidus* sp. strain DSPFs

Purification step	Total volume (mL)	Total activity (µmol min^−1^)	Total protein (mg)	Specific activity^*a*^ (µmol min^−1^ mg^−1^)	Fold purification	Yield (%)
Cell-free extract	28.8	50.7	268	0.19	1.0	100
Ammonium sulfate fractionation (30%–60%)	2.8	37.1	113	0.33	1.7	70
Affinity chromatography (2,4-DCP-Sepharose CL-4B)	10	24.8	10.8	2.29	12.1	49
Size exclusion chromatography (Enrich SEC 650)	3	3.2	1.1	2.92	15.4	6.2

^*a*^ Enzyme activity was measured using 24DCP with NADH as cofactor.

Large-scale reaction with purified 24DCPM, followed by reaction product analysis by thin layer chromatography (TLC), showed the formation of 3,5-DCC (R_f_ = 0.56) with NADPH as well as with NADH (Fig. S4B and C; Supplementary material). HPLC analysis of the reaction mixture corroborated TLC results, showing rapid depletion of substrate and formation of product with NADPH as a cofactor compared to NADH (Fig. S4D and E; Supplementary material).

Far-UV CD spectra of purified 24DCPM indicated a secondary structure of 57% helices and 16% *β*-sheets (Fig. S5A; Supplementary material). Purified 24DCPM was yellow in color, and the UV-visible spectrum showed absorption maxima peaks at 275, 380, and 458 nm, similar to authentic FAD (Fig. 4C). Further analysis revealed the presence of ~3.9 mol of FAD per mol of native enzyme (homotetrameric), suggesting the requirement of one FAD molecule per subunit of enzyme. The fluorescence emission spectra of purified 24DCPM showed maxima at 530 nm, which overlapped with the emission spectrum of the authentic FAD (Fig. 4D), confirming the presence of FAD as a prosthetic group.

### Kinetic properties of 2,4-DCPM

The initial velocity of 24DCPM increased with an increase in 2,4-DCP concentration up to 50 µM, but declined by 18% and 25% at 100 µM and 200 µM, indicating substrate inhibition at high concentration (Fig. 4E; Table 2). The Michaelis constant (*K*_m_) for 2,4-DCP with NADH was 3.1 µM, with a maximum velocity (*V*_max_) of 3.3 µmol min^−^¹ mg^−^¹ and a turnover number (*k*_cat_) of 14 s^−^¹ (Table 2). A similar inhibition pattern was observed with NADPH; however, ~5 times higher *V*_max_ (15.5 µmol min^−^¹ mg^−^¹ protein) and *k*_cat_ (66 s^−^¹) were observed as compared to NADH (Table 2). This suggests that 24DCPM exhibits better catalytic activity with NADPH as compared to NADH. The enzyme followed Michaelis-Menten kinetics with both NADH and NADPH, showing *K*_m_ values of 204.6 and 46.8 µM and *V*_max_ values of 5.2 and 7.2 µmol min^−^¹ mg^−^¹ protein, respectively (Table 2; Fig. 4F; Fig. S5B through E; Supplementary material). The *k*_cat_ for NADH and NADPH was 22 and 31 s^−^¹, respectively. The low *K*_m_ and high catalytic efficiency for NADPH indicate a higher affinity and suggest a preference for NADPH as a cofactor for 24DCPM. These results corroborate with previous studies, where 24DCPM from *Defluvibacter lusatiensis* S1, *Pseudomonas cepacia,* and *Cupriavidus necator* JMP134 was reported as a homotetrameric protein with a subunit molecular mass of 63–69 kDa (32, 57, 58). Similar to 24DCPM enzymes from model strain *C. necator* JMP134 (25), 24DCPM of strain DSPFs could use both NADH and NADPH as cofactors, with preference for NADPH over NADH (Table 2). It exhibits a higher *V*_max_ (7.2 µmol min^−1^ mg^−1^) and low *K*_m_ (46.8 µM) for NADPH compared to previously reported strains (23, 32, 58). Additionally, the *V*_max_ of 24DCPM with substrate (2,4-DCP) was significantly high (10-fold higher with NADPH) as compared to that of the model strain *C. necator* JMP134. This enhanced catalytic efficiency (high *V*_max_ and low *K*_m_) of 2,4-DCPM potentially contributes to the efficient degradation of 2,4-D observed in *Cupriavidus* sp. DSPFs compared to other bacteria.

**TABLE 2:** Kinetic properties of 2,4-dichlorophenol-6-monooxygenase from *Cupriavidus* sp. strain DSPFs

	*K*_m_ (µM)		*V*_max_ (µmol min^−1^ mg^−1^)		*K*_i_ (µM)		*K*_cat_ (s^−1^)		*K*_cat_/*K*_m_ (µM^−1^ s^−1^)	
	I^*a*^	II	I	II	I	II	I	II	I	II
2,4-DCP with NADH	3.1 ± 0.8^*b*^	3.7 ± 0.6	3.3 ± 0.4	3.4 ± 0.3	374 ± 80	287 ± 76	14 ± 1	14.4 ± 2	4.5 ± 0.6	3.9 ± 0.2
2,4-DCP with NADPH	10 ± 3	8.3 ± 2	15.5 ± 2	13.4 ± 3	268 ± 82	390 ± 56	66 ± 8	57 ± 13	6.6 ± 1	6.9 ± 1
NADH	205 ± 47	165 ± 36	5.2 ± 0.5	4.3 ± 0.2	–^*c*^	–	22 ± 2	18 ± 4	0.1 ± 0.01	0.11 ± 0.01
NADPH	46.8 ± 6	34 ± 6	7.2 ± 0.2	6.4 ± 0.3	–	–	31 ± 1	27 ± 2	0.7 ± 0.1	0.8 ± 0.1

^*a*^ Values are determined by using spectrophotometric (I) or polarographic (II) methods.

^*b*^ Values are presented as mean ± standard deviation.

^*c*^ ”–” indicates no inhibition observed.

### Plant growth enhancement and phytoprotection by strain DSPFs against toxicity of 2,4-D

**Fig 5:**
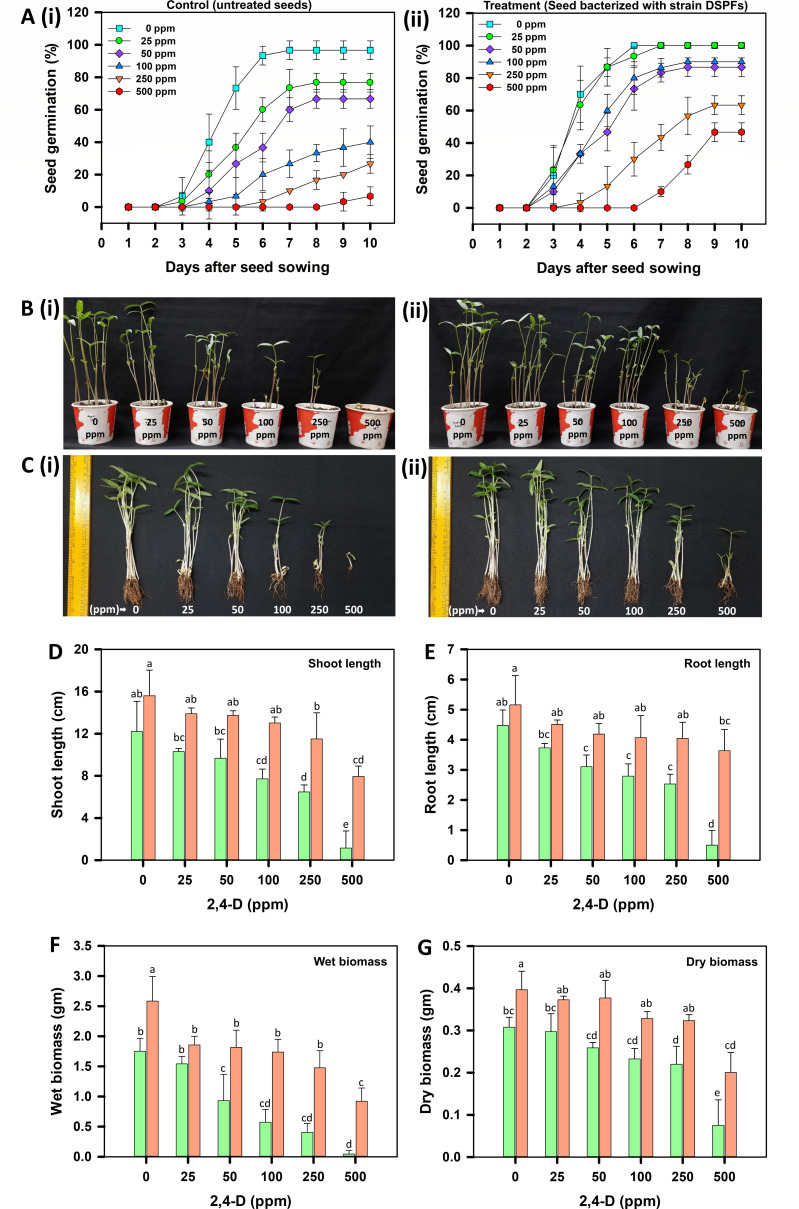
Toxicity of 2,4-D to mung bean (*Vigna radiata*) seedlings and phytoprotection (release of toxicity) by *Cupriavidus* sp. strain DSPFs in microcosm. (**A**) depicts the germination pattern of untreated seeds (control; Ai) and seeds bacterized with strain DSPFs (Aii) in the presence of different concentrations of 2,4-D (0, 25, 50, 100, 250, or 500 ppm). Each point represents the mean of percent germination, and error bars represent the standard deviation. (**B**) and (**C**) represent the differences in shoot and root length, respectively, of mung bean seedlings grown from (i) untreated seeds (control) and (ii) seeds bacterized with strain DSPFs in soil spiked with different concentrations of 2,4-D. Scales in images represent the scale bar (25 cm). The bar graphs in (**D**), (**E**), (**F**), and (**G**) depict the growth parameters measured for mung bean seedlings grown from untreated seeds (control; 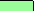) or seeds bacterized with strain DSPFs (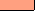) in the presence of different concentrations of 2,4-D. Columns in the bar graph represent the mean of shoot length (**D**), root length (**E**), wet biomass (**F**), and dry biomass (**G**) of mung bean seedlings on the 10th day after sowing. Error bars represent standard deviation. Different lowercase letters denote statistically significant differences among different treatments at *P* < 0.05 according to Tukey’s post hoc test (*n* = 3, *N* = 60).

Phytoprotective abilities of strain DSPFs were evaluated in microcosms spiked with 0, 25, 50, 100, 250, or 500 ppm of 2,4-D and sown with mung bean seeds (untreated or bacterized with strain DSPFs). All the assessed concentrations of 2,4-D were found to be toxic to the mung bean crop. At higher doses (100, 250, and 500 ppm), 2,4-D caused significant mortality (60–95%) of untreated seeds. Seed bacterization with strain DSPFs significantly enhanced germination to 85–90% in soils spiked with 50 and 100 ppm of 2,4-D and improved germination by 30–40% at 250 and 500 ppm (Fig. 5A). Significant decrease (*P* < 0.05) in shoot, root length (35–90%), and biomass (40–95%) was observed in untreated seedlings grown in 2,4-D spiked soil compared to non-spiked controls (Fig. 5B through G). Growth parameters of seedlings grown from bacterized seeds in contaminated soil were comparable to those in non-contaminated microcosms, indicating the ability of strain DSPFs to mitigate 2,4-D toxicity to crop plants.

**Fig 6:**
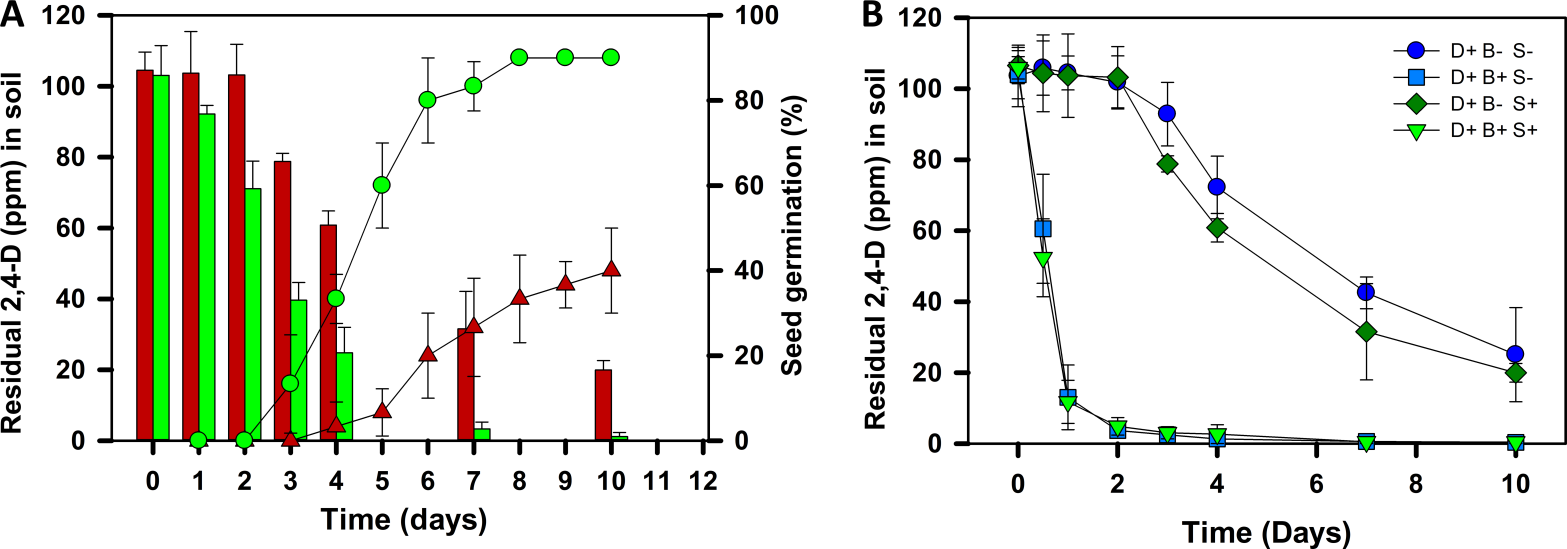
Degradation of 2,4-D in soil by *Cupriavidus* sp. strain DSPFs. (**A**) depicts the correlation between 2,4-D degradation and germination of mung bean (*Vigna radiata*) seeds bacterized with or without strain DSPFs in microcosms spiked with 2,4-D (100 ppm). Bars represent the mean of 2,4-D concentration in microcosms sown with untreated seeds (control; 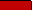) or bacterized seeds (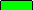) (*n* = 3, *N* = 9). Each point on the line represents the mean percent germination of untreated seeds (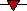) and bacterized seeds (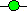) (*n* = 3, *N* = 90). (**B**) represents the degradation/removal of 2,4-D from 2,4-D contaminated soil by strain DSPFs. The soil in microcosms was spiked with 2,4-D (100 ppm; D+), inoculated with/without bacteria (B+/B−), and sown with/without mung bean seeds (S+/S−). D+ B− S−, soil spiked with 2,4-D (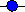); D+ B+ S−, soil spiked with 2,4-D and inoculated with strain DSPFs (10^7^ CFU g^−1^ of soil, 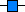); D+ B− S+, soil spiked with 2,4-D and sown with mung bean seeds (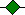), D+ B+ S+, soil spiked with 2,4-D, inoculated with strain DSPFs and sown with mung bean seeds (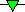). Each point on the line represents the mean of residual 2,4-D concentration in the soil, and error bars represent standard deviation (*n* = 3, *N* = 9).

The estimation of residual 2,4-D concentration in contaminated microcosms (spiked with 100 ppm of 2,4-D and sown with untreated seeds) revealed that ~25% of 2,4-D remained after 10 days, resulting in significant inhibition of seed germination (Fig. 6A). Contrarily, in the microcosms sown with bacterized seeds ~63% of 2,4-D was degraded within 3 days, and residual concentration of 2,4-D became negligible within 7 days (Fig. 6A). A strong correlation (r = 0.94, *P* = 0.0006) was observed between reduced 2,4-D levels and enhanced seed germination in microcosm sown with bacterized seeds. These results highlight the role of strain DSPFs in degrading 2,4-D from soil, thereby reducing the phytotoxicity of 2,4-D to crop plants.

Interestingly, seedlings grown from bacterized seeds in non-spiked soil showed enhanced growth (~30% increase in shoot/root length) and significant biomass increase (30–47%) as compared to control (untreated seedlings) (Fig. 5D through G). These results suggest the PGP potential of strain DSPFs, which could be attributed to various PGP traits possessed by this strain. It produced significantly higher IAA in LB medium supplemented with L-tryptophan (49.4 ± 3 µg mL^−^¹) compared to unamended medium (10 ± 0.3 µg mL^−^¹). It solubilized 6.5 ± 0.7 mg mL^−^¹ of phosphate in Pikovskaya’s broth and showed potassium solubilization on Aleksandrov’s agar medium with a solubilization index of 135 ± 5. Additionally, strain DSPFs produced 82 ± 4% units of siderophores in King’s B medium and 23.7 ± 2.4 µg mL^−^¹ of ammonia in peptone water after 72 h of incubation.

Reports on the release of the phytotoxicity caused by 2,4-D to crops are limited (16, 46). Xia et al. (16) reported that the presence of 2,4-D (50 and 100 ppm) in soil caused toxicity to maize seedlings that was mitigated by the inoculation of 2,4-D-degrading bacterium, *Achromobacter* sp. LZ35. However, strain LZ35 showed better phytoprotection at a lower dose, i.e., 50 ppm, as compared to a 100 ppm dose of 2,4-D (16). Phytotoxicity of 2,4-D (0.25 mM, i.e., ~55 ppm) to model plant *Arabidopsis thaliana* was effectively released by the inoculation of *Escherichia coli* BL-364 genetically engineered with *tfd*ABCDEF genes for complete degradation of 2,4-D (46). The ability of strain DSPFs to enhance crop plant growth and reduce the toxicity of 2,4-D broadens its applicability in sustainable agriculture for increased crop production.

### Bioremediation of 2,4-D contaminated soil by strain DSPFs

Bioremediation experiments were conducted in microcosms spiked with 100 ppm of 2,4-D (D+). The soil was inoculated (B+) with strain DSPFs (10^7^ CFU g^−1^ of soil) and either sown with mung bean seeds (S+) or left unsown (S−). Results demonstrated that strain DSPFs effectively removed over 80% of 2,4-D within 24 h with a degradation rate of 91 mg kg^−1^ d^−1^ (half-life = 0.59 day) and reached almost zero within 4 days, irrespective of seed presence (D+ B+ S− and D+ B+ S+) (Fig. 6B). Conversely, in uninoculated soil, a delay of around 3 days was observed, followed by slow degradation of 2,4-D by native microbiota, with a maximum rate of degradation reached up to 10 mg kg^−1^ d^−1^ (half-life = 9.2 days). Around 25% of 2,4-D remained even after 10 days of incubation (Fig. 6B). After the third day of incubation, ~1–5 ppm of 2,4-DCP (a metabolic intermediate in 2,4-D degradation) was detected in uninoculated microcosms, indicating incomplete degradation of 2,4-D and release of toxic metabolites in soil. Contrarily, no metabolites were detected in microcosms inoculated with strain DSPFs, suggesting that the strain degrades 2,4-D effectively in soil, making it a promising candidate for bioremediation of contaminated sites even at relatively high concentrations (100 ppm).

Exogenously applied bacteria often encounter environmental stresses in soil, which can negatively impact their survival and degradation efficiency. However, in this study, the bioaugmentation of contaminated soil with strain DSPFs resulted in the rapid degradation of 100 ppm 2,4-D within just 4 days. This suggests the strong adaptability of strain DSPFs to complex soil environments and highlights its notably fast detoxification capability as compared to previously reported bacterial strains (16, 26, 27). The concept of preventive bioremediation, introduced by Önneby et al. (59), involves the simultaneous application of pesticides and pesticide-degrading microorganisms to mitigate environmental contamination. This approach was further validated by Carles et al. (60) using 2,4-D as a model pesticide, proving its effectiveness as a promising strategy to reduce contamination from agricultural pesticides. For successful preventive bioremediation, microorganisms must exhibit a high degradation rate and minimal lag phase after inoculation. The rapid degradation of 2,4-D by strain DSPFs makes it an ideal candidate for preventive bioremediation, offering the potential to efficiently degrade herbicides in agricultural soils before they leach into and contaminate ground and surface water.

### Conclusion

Widespread use and persistence of 2,4-D in soils, along with its potential ecotoxicity and slow degradation under field conditions, there is a clear need for efficient bioremediation strategies. The 2,4-D degrading bacterial strain, *Cupriavidus* sp. strain DSPFs, was isolated from herbicide-impacted agricultural soil from India. Strain exhibits remarkable tolerance and efficiency in degrading high concentrations of 2,4-D, up to 3,000 ppm. The rate of biodegradation by this strain is higher than that of reported strains, including the model strain *C. necator* JMP134. Metabolite identification, whole-cell oxygen uptake, and enzyme activity studies indicated that strain DSPFs metabolizes 2,4-D via 2,4-DCP → 3,5-DCC → 2,4-dichloromuconic acid → central carbon pathway. The efficient degradation of 2,4-D by strain DSPFs can be attributed to the enhanced catalytic efficiency of enzyme 24DCPM. Strain has the ability to degrade a wide range of aromatic compounds and possesses metabolic diversity to degrade central aromatic intermediates such as catechol, gentisate, and protocatechuate. Strain DSPFs possess plant growth-promoting traits, including phosphate and potassium solubilization, production of phytohormone (IAA), ammonia, and siderophores. Seed priming with strain DSPFs resulted in enhanced germination and seedling growth of mung bean with mitigated phytotoxicity caused by 2,4-D. Inoculation of strain DSPFs (bioaugmentation) could effectively degrade 2,4-D in a soil microcosm spiked with 100 ppm of 2,4-D. This study revealed the multifaceted potential applications of strain DSPFs for (i) preventive bioremediation of herbicide (2,4-D) in agriculture to avoid ground and surface water contamination, (ii) effectively mitigating the phytotoxicity of herbicides, and (iii) enhancing plant growth and crop production.

## SUPPLEMENTAL MATERIAL

Supplemental figures and tables spectrum.00560-25-s0001.pdf Figures S1 to S5 and Tables S1 to S3.

ASM does not own the copyrights to Supplemental Material that may be linked to, or accessed through, an article. The authors have granted ASM a non-exclusive, world-wide license to publish the Supplemental Material files. Please contact the corresponding author directly for reuse.
