# Supplementary material for: Degradation of 2,4-D by plant growth-promoting Cupriavidus sp. DSPFs: role in mitigating herbicide toxicity in soil and enhancing crop production
Source: Microbiol Spectr. 2025 Sep 24;13(11):e00560-25. doi: 10.1128/spectrum.00560-25 (PMC12584615; doi:10.1128/spectrum.00560-25)
Supplement: Supplemental figures and tables — Figures S1 to S5 and Tables S1 to S3. [file spectrum.00560-25-s0001.pdf]

Supporting information

Degradation of 2,4-D by plant growth promoting *Cupriavidus* sp. DSPFs: Role in mitigating herbicide toxicity in soil and enhancing crop production

Sandesh E. Papade[1], Minhaaz Suhail[1], Om K. Bagwe[1] and Prashant S. Phale[1, *]

[1]Department of Biosciences and Bioengineering, Indian Institute of Technology-Bombay, Powai, Mumbai-400 076, India.

*Corresponding author: Dr. Prashant S. Phale (pphale@iitb.ac.in)

Supplementary Figures

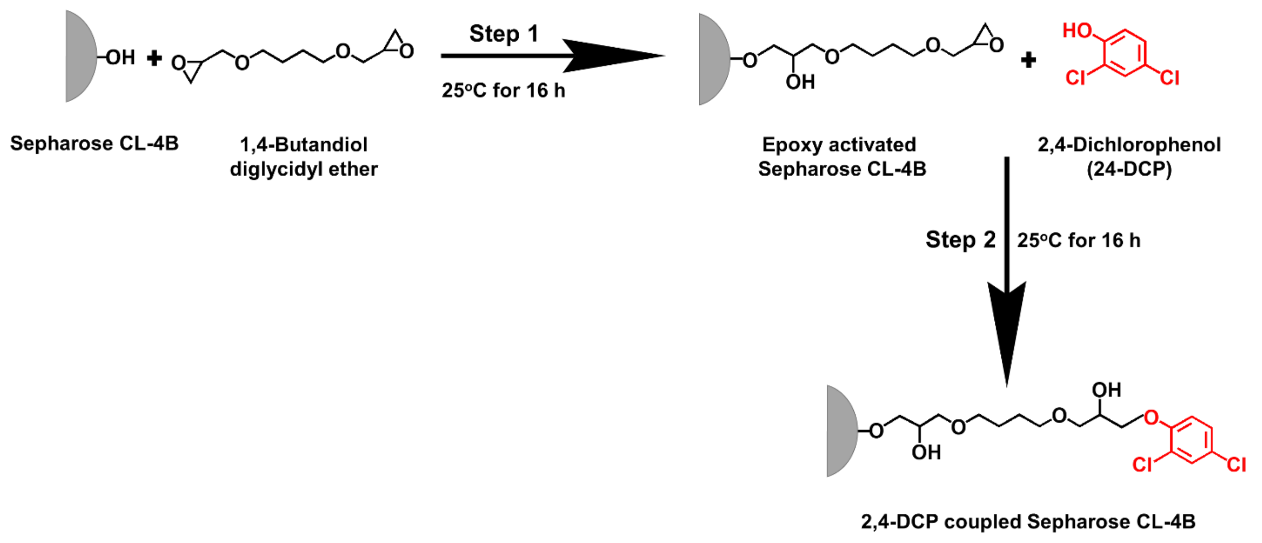

Figure S1: Preparation of 2,4-DCP-Sepharose CL-4B affinity matrix. **Step 1**: Sepharose CL-4B (5 g) was suspended in water (5 mL), 2 N NaOH (0.75 mL) and 1,4-butanediol diglycidyl ether (1 mL) and incubated at 25°C on to end-to-end shaker for 16 h. Excess 1,4-butanediol diglycidyl ether was removed by series of washing with water (7 mL), acetone (7 mL) and water (7 mL). **Step 2**: 2,4-DCP (50 mg) was gently mixed with of epoxy-activated Sepharose CL-4B in coupling solution (10 mL) *i.e.*, 0.1 M sodium carbonate (pH 10.5) and incubated at 25°C on to end-to-end shaker for 16 h. After coupling, excess ligand was removed by series of washing with coupling solution, water, 0.1 M borate buffer (pH 8.0) and then with 0.1 M acetate buffer (pH 4.5). Uncoupled epoxy groups were blocked with ethanolamine (10 % v/v) in distilled water (7 mL). The affinity matrix was washed thoroughly with Milli-Q water followed by phosphate buffer (50 mM, pH 7.5) and suspended in the same buffer till further use (Radjendirane et al., 1991).

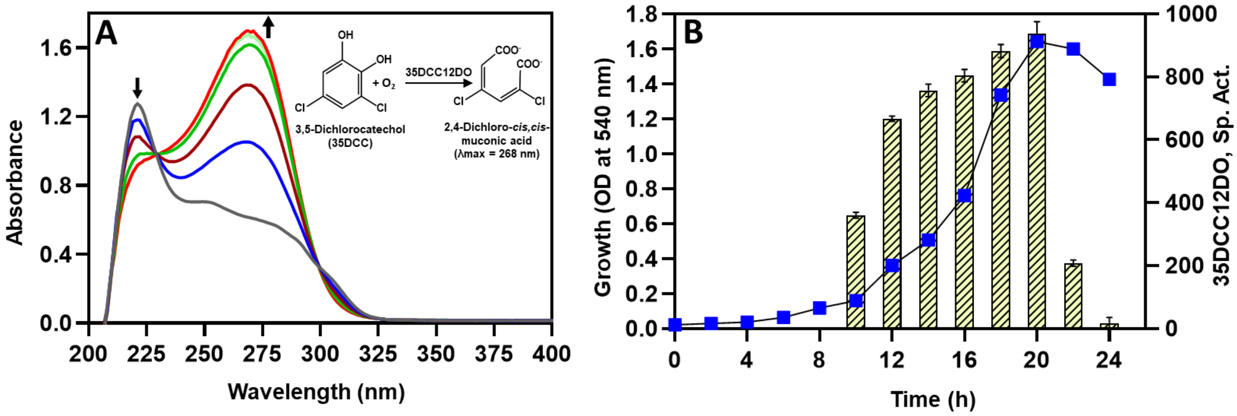

Figure S2: The activity of 3,5-dichlorocatechol-1,2-dioxygenase (35DCC12DO) from *Cupriavidus* sp. strain DSPFs. Panel **A** represents time-dependent spectral changes for conversion of 3,5-dichlorocatechol to 2,4-dichloro-*cis*,*cis*-muconic acid. Time-dependent spectral changes were recorded with CFE prepared from cells grown on 2,4-D (0.2 %, w/v). The enzyme reaction was scanned from 200-400 nm every 1 min interval for 10 cycles with 3,5-dichlorocatechol (100 μM) as the substrate. The spectral scan showed decrease in the absorbance at 220 nm (down arrow) and increase in the absorbance at 268 nm (up arrow) indicating the conversion of 3,5-dichlorocatechol to 2,4-dichloro-*cis*,*cis*-muconic acid. Inset displays reaction catalysed. Panel **B** depicts the growth (-■-) dependent specific activity (Sp. Act) of 35DCC12DO (▨) of strain DSPFs grown on 2,4-D (0.2 %, w/v).

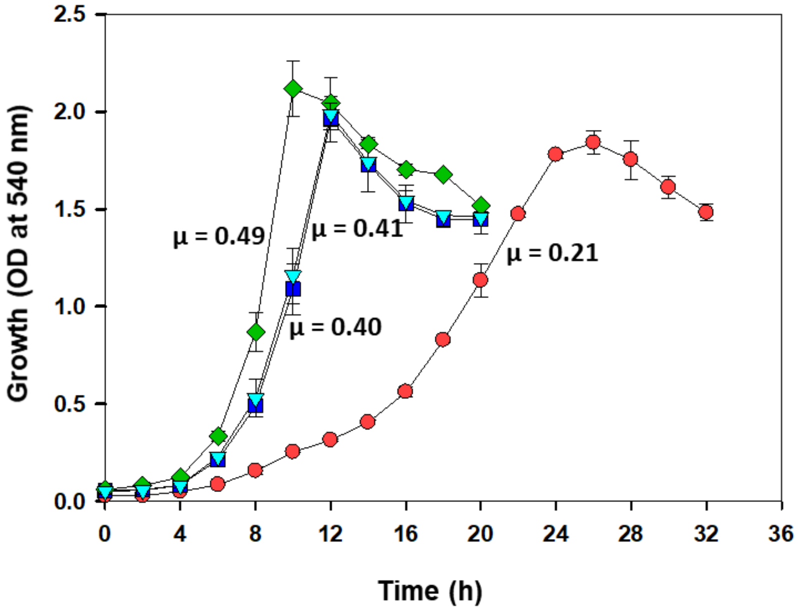

Figure S3: Growth profile of *Cupriavidus* sp. strain DSPFs on benzoate (-●-), 2-hydroxybenzoate (salicylate; -■-), 3-hydroxybenzoate (-◆-) and 4-hydroxybenzoate (-▽-). Culture was grown on either of the aromatics (0.1 %, w/v) as sole carbon source in MSM (pH 7.5) at 30°C. The specific growth rate, μ (h^{-1}) is depicted next to respective growth profile. Experiments were performed independently at least three times and data is presented as arithmetic mean with standard deviation.

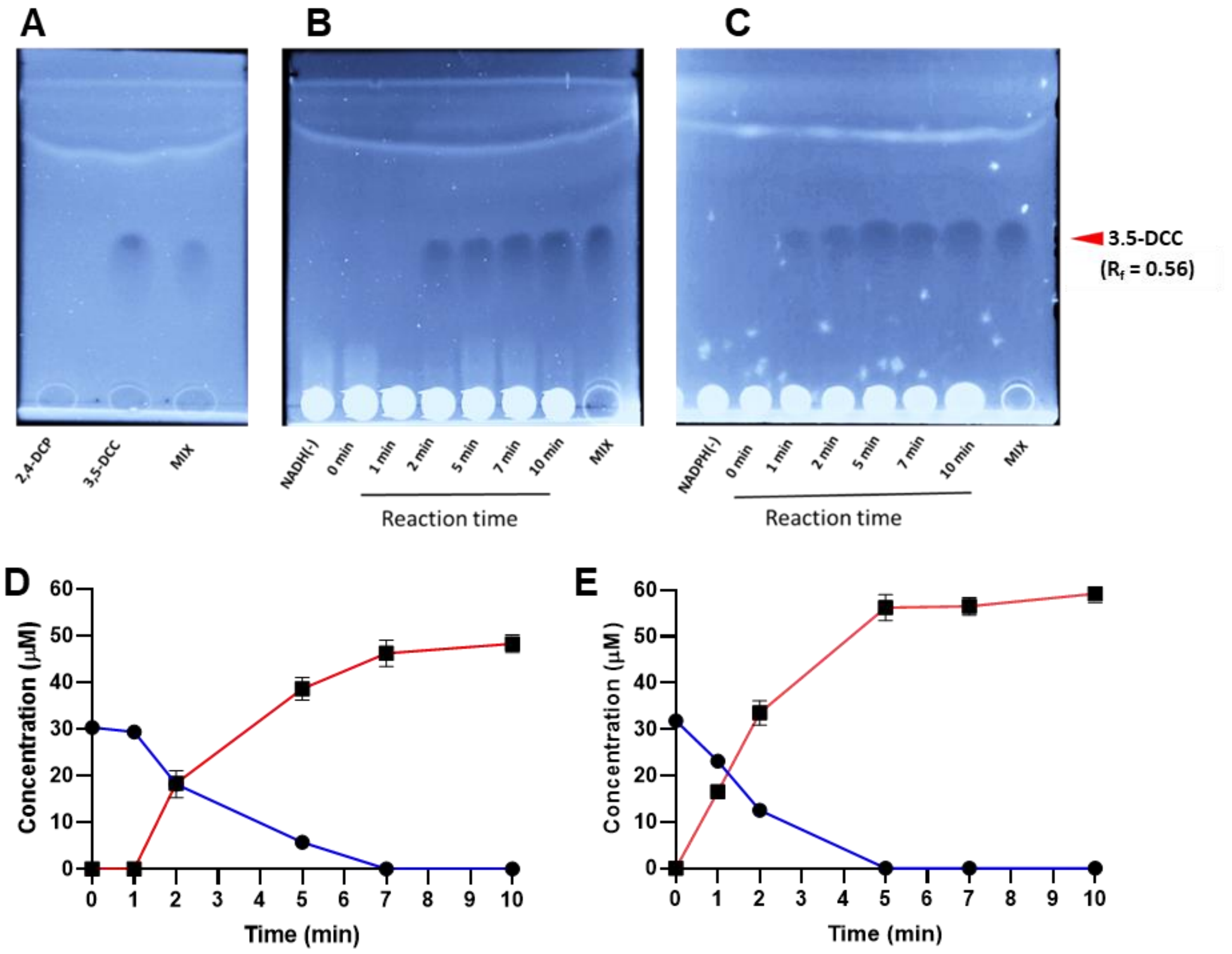

Figure S4: Analysis of reaction product of 2,4-dichlorophenol-6-monooxygenase (24DCPM) from *Cupriavidus* sp. strain DSPFs. Image in panel **A** depicts chromatogram of authentic compounds resolved by TLC as standard/reference: *Lane 1*, substrate (2,4-DCP), *Lane 2,* product (3,5-DCC) and *Lane 3*, mixture of 2,4-DCP and 3,5-DCC. Panel **B** and **C** depict analysis of reaction product (3,5-DCC) formation using NADH **(B)** and NADPH **(C)** as the cofactor. NADH(-) and NADPH(-) in panel **B** and panel **C** depicts control reaction without cofactor, respectively. Subsequent lanes depict reaction time intervals (0, 1, 2, 5, 7 and 10 minutes). Last lane (MIX) contain mixture of authentic 2,4-DCP and 3,5-DCC as a reference. Panel **D** and **E** depict the HPLC analysis of reaction mixture indicating decrease in substrate (2,4-DCP) concentration (–●–) and formation of product (3,5-DCC; –■–) using NADH **(D)** or NADPH **(E)** as the cofactors.

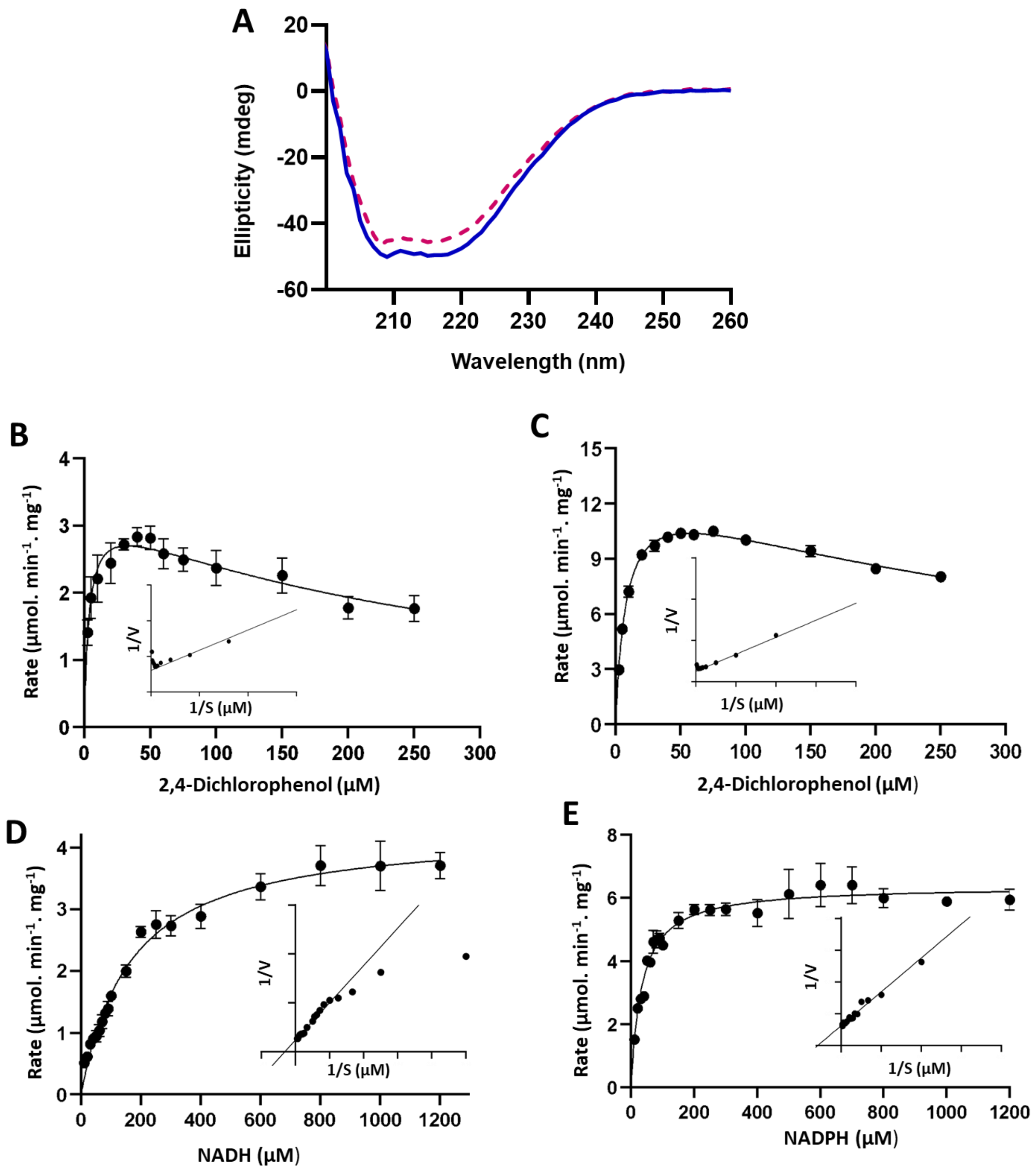

Figure S5: Spectroscopic and kinetic properties of 2,4-dichlorophenol-6-monooxygenase (24DCPM) from *Cupriavidus* sp. strain DSPFs. Panel **A** depicts Far-UV CD spectra of 24DCPM (1 µM, solid line) and 24DCPM with 2,4-DCP (50 µM, dashed line) in potassium phosphate buffer (50 mM, pH 7.5). Panel **B** and **C** depicts plots of initial velocity (*v,* determined polarographically) versus [*S*] for 24DCPM with NADH **(B)** and NADPH **(C)** as the co-factors. Panel **D** and **E** represent Michaelis-Menten plot for varying concentrations of NADH **(D)** and NADPH **(E)**.

Supplementary Tables

Table S1: Colony characteristics and biochemical properties of *Cupriavidus* sp. strain DSPFs.

**Characteristics/tests**	**Observation**
**Colony and cell Characteristics**	
Colony size	2-3 mm
Configuration/shape	Circular
Colour	Cream-white
Margin	Entire
Opacity	Opaque
Elevation; Surface	Convex; Smooth
Pigment production	No pigments
Gram’s reaction	Gram negative
Cell shape	Rod
Motility	Motile
**Physiological and Biochemical tests**	
Oxidase test	Positive
Catalase	Positive
Arabinose utilization	Negative
Glucose utilization	Negative
Galactose utilization	Negative
Fructose utilization	Positive
Rhamnose utilization	Negative
Maltose utilization	Negative
Sucrose utilization	Negative
Lactose utilization	Negative
Glycerol utilization	Positive
Adonitol utilization	Negative
Mannitol utilization	Negative
Sorbitol utilization	Negative
Citrate utilization	Positive
α-Ketoglutarate utilization	Positive
Succinate utilization	Positive
Fumarate utilization	Positive
Malate utilization	Positive
Acetate utilization	Positive

Table S2: Stability of 2,4-D degradation phenotype in *Cupriavidus* sp. strain DSPFs.

**Growth conditions**	**Concentration (μg/mL)**	**Colonies screened**	**Colonies grown on 2,4-D**	**Degradation Phenotype Stability (%)**
**Luria broth (LB)**	-	820	820	100
**LB + Ethidium bromide**	10	541	541	100
	25	514	514	100
	50	550	550	100
**LB + Acridine orange**	10	494	494	100
	25	497	497	100
	50	617	617	100

Table S3: Specific activities of different enzymes involved in the degradation of benzoate and hydroxybenzoates by *Cupriavidus* sp. strain DSPFs.

**Enzymes***	**Specific activity (nmol min^{-1} mg^{-1} protein) in CFE of DSPFs cells grown on**			
	**Benzoate**	**2-Hydroxybenzoate (Salicylate)**	**3-Hydroxy-benzoate**	**4-Hydroxy-benzoate**
**C12DO**	**157 ± 5**[$]	6.9 ± 2	ND[#]	ND
**C23DO**	0.4 ± 0.1	1.2 ± 1.1	ND	ND
**P34DO**	10 ± 3	ND	7.8 ± 6.9	**251 ± 7**
**P45DO**	0.3 ± 1	ND	0.3 ± 0.2	3.8 ± 3.4
**G12DO**	2.5 ± 1	**261 ± 10**	**348 ± 7**	NA

*C12DO, catechol 1,2-dioxygenase; C23DO, catechol 2,3-dioxygenase; P34DO, protocatechuate 3,4-dioxygenase; P45DO, protocatechuate 4,5-dioxygenase; G12DO, gentisate 1,2-dioxygenase.

[$] Values in bold indicates involvement of enzyme in the degradation of respective aromatic compound.

[#] ND, not determined.
